# A Case of Idiopathic Recurrent Spontaneous Bladder Rupture

**DOI:** 10.1155/2021/6615817

**Published:** 2021-08-30

**Authors:** Reid Bartholomew, Mentor Ahmeti

**Affiliations:** ^1^Department of Surgery, University of North Dakota, Grand Forks, ND 58202, USA; ^2^Trauma and Acute Care Surgery, Sanford Medical Center, Fargo, ND 58104, USA

## Abstract

**Background:**

A female patient presented four years following spontaneous bladder rupture with a recurrent spontaneous bladder rupture. *Summary*. Urinary bladder rupture is a condition usually caused by trauma or surgical instrumentation. Spontaneous bladder rupture is a much more uncommon condition and is associated with intoxication, radiation, stricture, or neurogenic bladder. We describe a case of a 40-year-old woman with a history of three caesarian sections with an idiopathic recurrent spontaneous bladder rupture. Originally, she presented with one day of worsening severe abdominal pain. CT showed possible ischemic bowel. She was taken to the operating room (OR) and found to have a bladder rupture. This was repaired, and she did well postoperatively. Four years later, she presented to the emergency department (ED) with one week of worsening abdominal pain that became severe acutely. Given that she had a similar issue four years prior the patient was suspicious, her bladder was again ruptured. CT cystogram showed contrast extravasation into the peritoneum. The patient was taken urgently to the operating room for an open repair of the bladder rupture. She did well following the procedure.

**Conclusion:**

Spontaneous bladder rupture is a surgical emergency and should be in the differential diagnosis of any patient with peritonitis with elevated creatinine and free intraperitoneal fluid. This diagnosis should especially be considered if the patient has a history pelvic radiation, neurogenic bladder, or intoxication. We submit that a history of multiple pelvic surgeries should be included in this list. CT cystogram is the diagnostic test of choice. Operative repair is generally the treatment for this condition.

## 1. Introduction

Spontaneous bladder ruptures are usually associated with intoxication, radiation, stricture, cancer, or a neurogenic bladder [1–10]. Rarely, they can be idiopathic [11–13] or recurrent [14–19], and a recurrent idiopathic spontaneous bladder rupture has only been described once [20]. Historically, they have been associated with significant morbidity and mortality [5, 7–9, 17]. Diagnosis is made with CT cystography or traditional cystography [20, 21]. Spontaneous bladder ruptures are usually treated with operative repair [6–8, 12], but occasionally have been treated nonoperatively with Foley catheter placement [21].

## 2. Case Presentation

We present a case of recurrent spontaneous bladder rupture in a 40-year-old Caucasian woman with a history of three uncomplicated caesarian sections and an uncomplicated hysterectomy eight years prior. Four years prior to presentation at our facility, she was hospitalized with 1 day of increasingly severe abdominal pain and two weeks of dysuria and foul smelling urine. She denied changes in urinary frequency, urgency, nocturia, weak stream, intermittency, straining, or sensation of incomplete emptying; international prostate symptom score (IPSS) of 0; no trauma, blow to abdomen, falls, or intoxication prior to presentation. CT of the abdomen and pelvis showed free pelvic fluid and bowel wall edema concerning for small bowel perforation. She was taken to the operating room (OR) for an exploratory laparotomy, where a spontaneous rupture involving the dome of the bladder was discovered. Her bladder was repaired at this time, and she did well postoperatively.

**Figure 1:**
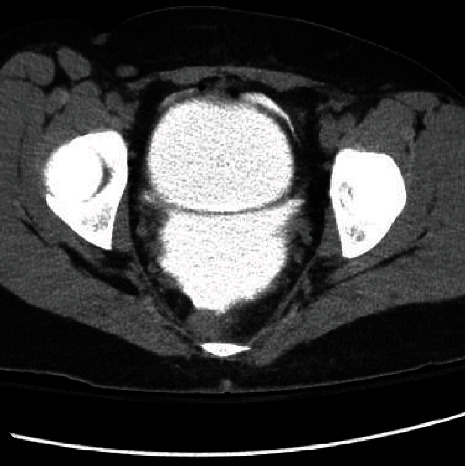
CT cystogram showing contrast extravasation into peritoneum and a small amount of free peritoneal air.

Four years later, she presented to the emergency department (ED) with a one-week history of mild lower abdominal pain that acutely worsened to severe pain in the middle of the night. She had also been having nausea, vomiting, dysuria, and urinary retention more than half of the time. She denied changes in urinary frequency, urgency, nocturia, weak stream, intermittency, or straining and IPSS of 4. She denied any recent trauma, blow to abdomen, falls, or intoxication. She never had radiation and does not have a neurogenic bladder. On arrival to the ED, she was hemodynamically stable. The patient stated that her symptoms were nearly identical to her previous spontaneous bladder rupture. Physical exam revealed severe lower abdominal tenderness to palpation, distention, guarding, and peritoneal irritation. A Foley catheter was placed and had return of a small amount of clear urine output. Labs were obtained and showed leukocytosis of 11.7 × 10^9^/L, creatinine of 1.09 mg/dL, lactate of 3.8 mmol/L, and a urinalysis with trace blood and leukocytes. A CT cystogram was obtained and showed a bladder inflated with contrast and a small amount of air in the lumen. Contrast extravasation within the peritoneum was also seen, along with a small amount of free intraperitoneal air (Figure 1). CT cystogram also showed normal bladder capacity, no diverticulum, and no signs of neurogenic bladder such as Christmas tree. At this point, the patient was taken emergently to the OR.

**Figure 2:**
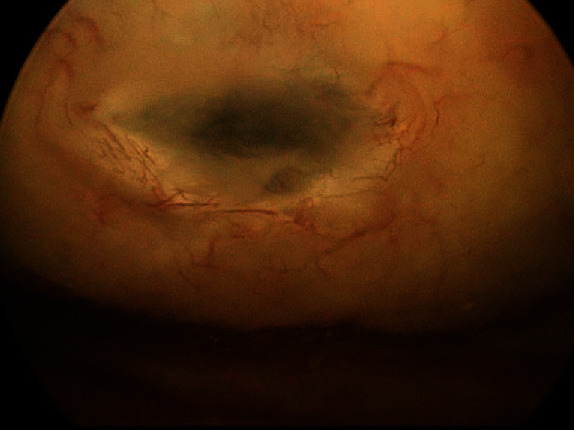
Intraoperative flexible cystogram—rupture of bladder dome.

Upon arrival to the OR, general anesthesia was induced. The patient was placed in the lithotomy position and prepped and draped in the usual sterile fashion. Cystoscopy was performed showing a small bladder opening at the dome (Figure 2). The bladder in this area appeared to be very thin.

Suprapubic midline incision was made using the previous laparotomy scar. Upon entry to the abdomen, minimal adhesions were encountered none identified in the pelvis or around the bladder. A one cm bladder rupture was noted at the dome of the bladder. The area around the rupture was noted to be thin, but the rest of the bladder appeared normal. The bladder rupture was repaired in two layers and then filled with 250 cc sterile saline with no leak noted. The suture line was then buttressed with an omental flap, and a 15 French Jackson-Pratt drain was placed in pelvis. The abdomen was then closed. The patient tolerated the procedure well.

**Figure 3:**
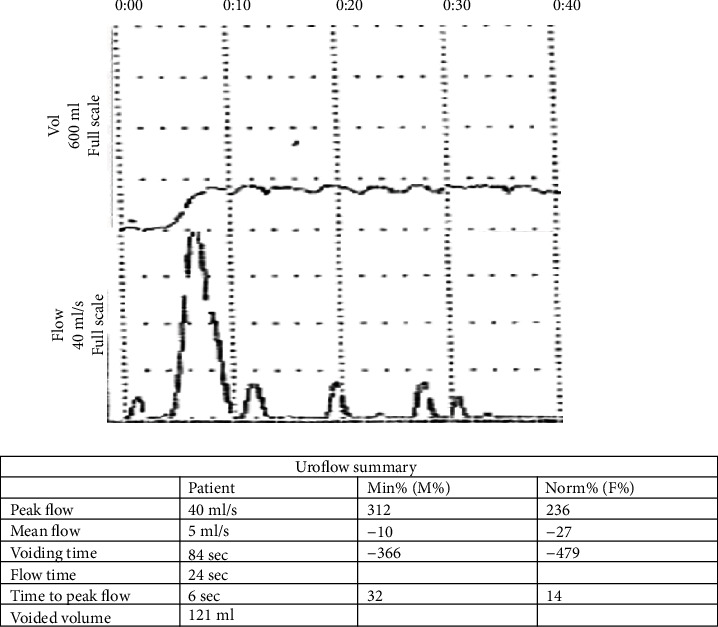
Postoperative urodynamic cystometry.

The patient had an uneventful recovery. The Jackson-Pratt drain was removed on postoperative day three. She was discharged on postoperative day four with a Foley catheter in place. Three weeks after discharge, she had a cystogram performed which demonstrated no leak. The Foley catheter was removed at this time. Further outpatient workup showed complete bladder emptying. Urodynamic cystometry was performed and revealed normal bladder pressures during both filling and voiding without detrusor instability (Figure 3). Pathology came back as focally ossified bladder mucosa without atypia. Since then, she has done very well and has had no other issues with her bladder.

## 3. Discussion

Spontaneous bladder rupture is uncommon (1 : 126,000) [5, 17] but well described in literature. It is classified as either intraperitoneal or extraperitoneal, with intraperitoneal being more common [8, 21]. Most frequently, spontaneous rupture happens at the dome or the posterior wall of the bladder [7, 8]. Recurrent bladder ruptures [14–19] and idiopathic ruptures are even more rare [11–13]. A recurrent idiopathic spontaneous bladder rupture, as with our patient, has only been described once in literature [20].

Expeditious diagnosis and emergent surgery is essential for good outcomes [1]. Most spontaneous bladder ruptures are not idiopathic, but are associated with a history of radiation, neurogenic bladder, or intoxication [1–10, 17, 21]. Idiopathic spontaneous bladder ruptures are unusual [11–14], and because of this, the diagnosis can be challenging. Patients typically present with findings similar to bowel perforation such as impaired mental status, severe abdominal pain, nausea, vomiting, and peritonitis on physical exam [2, 5, 7–10, 12, 17]. Spontaneous bladder rupture should be included in the differential diagnosis for any patient that presents with these findings [11]. Given that our patient had an elevated IPSS score prior to her second bladder perforation, increasing IPSS score is a useful tool to help predict subsequent rupture. Elevated serum creatinine can also be a diagnostic clue as longstanding intraperitoneal urine creatinine after rupture will be reabsorbed into the serum [3, 7–9]. Diagnosis can be made with CT cystogram or a traditional cystogram [20, 21]. Operative repair is almost always the treatment [6–8, 12], but there are rare cases where patients can be treated nonoperatively with Foley catheter drainage. The urinary bladder should be repaired in two layers with well-vascularized tissue [7, 8]. A Foley catheter should remain in place for at least two weeks following operative intervention [11]. Historically, the mortality of spontaneous bladder rupture approached 25-56% [5, 7–9, 17] but has most likely decreased due to higher quality imaging and better management of sepsis and electrolyte derangements [2, 3, 7, 14]. A missed diagnosis leading to a delay in treatment dramatically increase mortality [2, 3, 5, 14].

While diagnosis is often difficult, in our patient, it was less of a diagnostic mystery given the recurrent nature of her condition and the patient's insistence that her symptoms were identical to her previous bladder rupture. Prompt diagnosis in our patient led to early imaging and expedient surgical repair, resulting in an optimal outcome. We postulate that her history of numerous pelvic surgeries may have caused scar tissue formation around the dome of the bladder. This would lead to rigidity or weakness in the bladder wall leading to recurrent perforation of her bladder despite the fact that no adhesions were noted in this area nor any surrounding abnormalities during the operation.

## 4. Conclusion

Spontaneous bladder rupture is a surgical emergency. We present a case of recurrent spontaneous bladder rupture four years following spontaneous bladder rupture. This case highlights the need for spontaneous bladder rupture to be included in the differential diagnosis for any patient with peritonitis and elevated creatinine with a history of pelvic radiation, neurogenic bladder, or intoxication. We submit that given this case, previous pelvic surgery should be added to the aforementioned list.

## Data Availability

No datasets were generated or analyzed during the current study.
